# MBS: a genome browser annotation track for high-confident microRNA binding sites in whole human transcriptome

**DOI:** 10.1093/database/baad015

**Published:** 2023-04-22

**Authors:** Walter Arancio, Nicolina Sciaraffa, Claudia Coronnello

**Affiliations:** Institute for Biomedical Research and Innovation (IRIB), National Research Council (CNR), via Ugo la Malfa, 153, Palermo 90133, Italy; Advanced Data Analysis Group, Ri.MED Foundation, via Bandiera, 11, Palermo 90133, Italy; Advanced Data Analysis Group, Ri.MED Foundation, via Bandiera, 11, Palermo 90133, Italy; National Center for Gene Therapy and Drugs based on RNA Technology, via Bandiera, 11, Palermo 90133, Italy

## Abstract

MicroRNAs (miRNAs) are small non-coding ribonucleic acids (RNAs) that play a role in many regulatory pathways in eukaryotes. They usually exert their functions by binding mature messenger RNAs. The prediction of the binding targets of the endogenous miRNAs is crucial to unravel the processes they are involved in. In this work, we performed an extensive miRNA binding sites (MBS) prediction over all the annotated transcript sequences and made them available through an UCSC track. MBS annotation track allows to study and visualize the human miRNA binding sites transcriptome-wide in a genome browser, together with any other available information the user is interested in. In the creation of the database that underlies the MBS track, three consolidated algorithms of miRNA binding prediction have been used: PITA, miRanda and TargetScan, and information about the binding sites predicted by all of them has been collected. MBS track displays high-confident miRNA binding sites for the whole length of each human transcript, both coding and non-coding ones. Each annotation can redirect to a web page with the details of the miRNA binding and the involved transcripts. MBS can be easily applied to retrieve specific information such as the effects of alternative splicing on miRNA binding or when a specific miRNA binds an exon–exon junction in the mature RNA. Overall, MBS will be of great help for studying and visualizing, in a user-friendly mode, the predicted miRNA binding sites on all the transcripts arising from a gene or a region of interest.

**Database URL** https://datasharingada.fondazionerimed.com:8080/MBS

## Introduction

MicroRNAs (miRNAs) are small non-coding ribonucleic acids (RNAs), usually 20–24 nucleotides long, that play a role in many regulatory pathways in eukaryotes. miRNAs usually exert their functions by binding mature messenger RNAs (mRNAs) at their 3ʹ end in a region that does not code for protein called 3ʹ untranslated region (3ʹUTR). *In vivo*, miRNA binding and the following regulatory events are mediated by specific ribonucleic complexes. The most studied complex is the RNA-induced silencing complex (RISC), whose main activity is to downregulate the translation of the target transcripts. Noteworthy, (i) miRNAs can and do bind mRNA regions different from the 3ʹUTR, (ii) the complexes that make use of miRNAs can be different from the canonical RISC and (iii) miRNAs possess other roles apart from the inhibition of translation. For a detailed review on miRNA functions, please refer to dedicated literature (1–5).

The main issue in studying miRNA target regions is the difficulty to collect experimentally validated binding regions. This is usually done in a case-by-case approach, where the binding of a specific miRNA to a specific portion of an mRNA is hypothesized and then validated, while transcriptome-wide approaches have been rarely pursued (6).

By all these reasons, the prediction of the binding targets of the endogenous miRNAs is crucial to understand the processes they are involved in. Several algorithms and tools have been developed with this purpose in mind. They are generally based on base pair matching between miRNA and target regions of mRNA. One of the main issues of this approach is that miRNAs require incomplete matching with their targets, apart from a region of 6–8 nucleotides near the 5ʹ end of the miRNA called ‘seed’, so a vast majority of tools refine their predictions by making use of the calculation of binding energy, quantifying how much the binding site (BS) is evolutionary conserved or taking into account the relative expression of miRNA and mRNA in specific cells or tissues. Noteworthy, some tools and experimental approaches make a combinatorial use of all those parameters to better predict miRNA targets (7, 8). To complicate this scenario, each miRNA can bind many mRNAs, and each mRNA can be bound by several miRNAs. When miRNAs are in limiting amounts, different mRNAs can compete for the shared cellular pool of miRNAs acting as competing endogenous RNA or ceRNA, which represents a further layer of post-transcriptional regulation, also in pathological conditions such as viral infections, where abundant viral RNAs come into play (9–12).

In the creation of the database that underlies the MBS track, we took advantage of three consolidated algorithms for miRNA binding prediction: PITA (13), miRanda (14) and TargetScan (15).

PITA evaluates the accessibility of the miRNA target site: it estimates the difference between the free energy gained by the system from miRNA binding and the cost of opening the mRNA target region, considering also the entire miRNA sequence.

miRanda uses three properties to predict the target sequences for each miRNA: (i) sequence complementarity using a local alignment algorithm, (ii) free energies of the miRNA-target complexes and (iii) how much the target sites are evolutionary conserved.

TargetScan focuses on the presence of seed sequences but can consider accessory parameters such as pairing contribution outside the seed region, nucleotide content of the mRNA near the binding site, the position of the binding site in the target transcript (e.g. how much it is near the 3ʹUTR) and the conservation of seed region.

With the purpose in mind to develop a tool that can help researchers to study and visualize the miRNA binding sites transcriptome-wide in human, we create the underlying database and develop the MBS annotation track that allows to navigate human transcriptome in genome browsers such as UCSC (16).

The track displays high-confident miRNA binding sites retrieved by all the three algorithms in each annotated transcript sequence. This permits to obtain information and visualize the effect of alternative splicing on miRNA binding and even when a specific miRNA binds an exon–exon junction in the mature mRNA. Moreover, the analysis is performed on the whole length of mature transcripts, not only the 3ʹUTR, and considers the whole transcriptome, comprehending also the non-coding transcripts and not only the mature mRNAs.

Overall, MBS will be of great help for studying and visualizing the predicted miRNA binding sites of the transcripts arising from a gene or a region of interest.

## Methods

### Transcript sequences preprocessing

Human transcript sequences have been downloaded from Ensembl (17) on human GRCh38.p13 by Ensembl BioMart at https://www.ensembl.org/info/data/biomart/index.html retrieving the collection of all whole length mature transcripts (complementary deoxyribonucleic acid sequences). Transcripts alignment on the genome has been retrieved with the transcriptToGenome R function in the ensembldb library (18), by using the annotation hub ‘AH98047’. We considered only the transcripts confirmed to be aligned to canonical chromosomes. Therefore, we analyzed 225,291 transcripts.

### miRNA binding site prediction

The sequences of mature human miRNAs have been downloaded from miRBase (19), version 22. So far, 2656 miRNAs have been included in the analysis.

PITA (13), miRanda (14) and TargetScan (15) algorithms have been run using all miRNAs in collection against each mature transcript of whole human transcriptome. We selected as predicted binding sites the ones that satisfy the following conditions, also used in ref (20):

- miRanda: binding energy ≤−20 kcal/mol and score ≥140
- PITA: ΔΔE ≤−10 kcal/mol
- TargetScan: binding site type = 8mer-1a, 7mer-1a or 7mer-m8.

Only the binding sites predicted by all the three algorithms, with a BS sequence overlap of at least six nucleotides, have been retained in the database as high-confidence predicted targets. So far, 6,455,489 BS have been selected.

### MBS track description

The information about the predicted BS has been assembled in a single bedDetail file reporting the localization of the predicted binding sites on the chromosomal coordinates. Each BS is labeled with the name of the miRNA involved. If more than one miRNA is involved in exactly the same binding region, an asterisk is added to the label, and the list of all the miRNAs involved can be found in the detail page. Each predicted binding site is also associated with a link to an external web page, where more information regarding the predictions of the binding sites is shown. When the same BS is shared by multiple transcripts of the same gene and/or miRNAs, it is annotated as a single item, and the external web page includes the information on all the transcript/miRNAs pairs involved.

### System description

**Figure 1.**
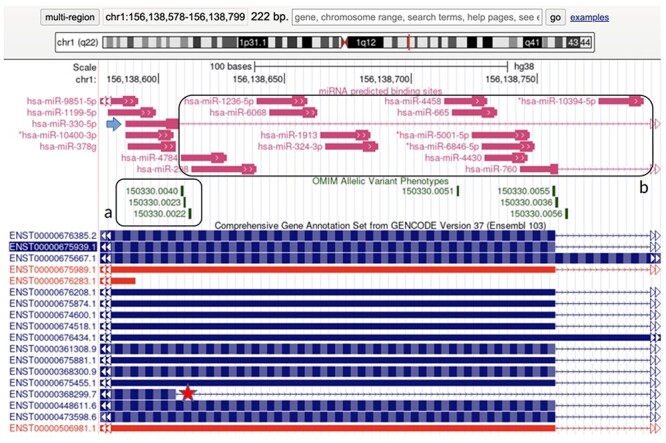
Visualization of a portion of the *LMNA* gene in UCSC genome browser (http://genome.ucsc.edu). (a) The vast majority of the causative mutations for HGPS cluster in this splicing junction. The star points out the ENST00000368299 transcript, the *LMNA* splicing form lacking 150 nucleotides, typical of HGPS. The arrow points out the BS predicted for hsa-miR-330-5p which binds specifically the aberrant splicing form involved in HGPS only. (b) miRNAs that do not recognize the aberrant splicing form involved in HGPS.

The MBS track can be displayed on a genome browser, typically the UCSC genome browser (16). Here, the user can navigate to the gene(s) or region(s) of interest. The custom MBS track can be displayed together with other information of interest, either embedded in the genome browser itself—such as annotations on mapping, gene and pseudogenes predictions, phenotypes, gene expression, regulation events and so on—or retrieved from other custom tracks if the need arises for a specific study. An example of display is found in Figure 1.

**Figure 2.**
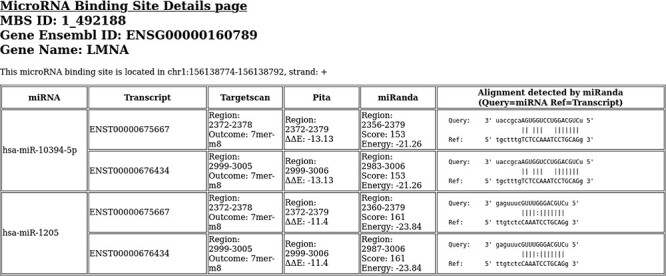
An example of binding site details html page. This binding site is located in *LMNA* gene.

By clicking on a specific binding site of MBS, it redirects to a web page generated by the UCSC genome browser that recapitulates the information of the selected binding site. In this page, an Outside Link can be clicked to be redirected to an external static web page with greater details on the miRNA binding site and the results of the algorithms that have predicted it (Figure 2).

### Database content

The information used to compile the MBS track can be retrieved in two different formats: (i) bedDetail file and the html files with extra information, both of them designed to be suitable for UCSC browser; (ii) csv file, delimiter = ‘;’.

The bedDetail file is designed as required by the UCSC framework, and it contains 14 tab-separated columns, showing the following information: (i) chromosome name; (ii) the starting position of the BS in the chromosome; (iii) the ending position of the BS in the chromosome; (iv) the name of the miRNA involved; (v) a score assigned to the BS (not used); (vi) the strand; (vii) equal to (ii); (viii) equal to (iii); (ix) an RGB (Red, Green, Blue code) value to set the color of the track line of the BS (set to 255, 255, 0); (x) the number of blocks involved in the BS (1—if the BS is fully contained in one exon, 2—if the BS is located across two exons); (xi) a comma-separated list of the block sizes; (xii) a comma-separated list of the block starts; (xiii) a unique code identifying the external link with detailed information and (xiv) a long description, currently used to list all the miRNAs involved in the BS.

Each html file visualizes information about the miRNAs/transcripts involved in the associated BS. It shows a table with six columns containing following information: (i) the miRNA involved; (ii) the transcript involved; (iii) Targetscan prediction details, i.e. the range of the binding region within the transcript and the prediction outcome; (iv) PITA prediction details, i.e. the range of the binding region within the transcript and the computed ΔΔE; (v) miRanda prediction details, i.e. the range of the binding region within the transcript and the computed binding energy and score and (vi) a schematization of the alignment between the miRNA and the transcript as detected by miRanda.

Finally, the csv file summarizes all the information contained in the bedDetail and html files in a tabular format. It is a semicolon-separated file with 14 columns containing the following information: (i) the Ensembl ID of the transcript involved in the BS, (ii) the Ensembl ID of the gene involved in the BS, (iii) the gene name, (iv) the miRNA involved in the BS, (v–vi) the range of the binding region within the transcript, (vii) chromosome name, (viii) strand, (ix–x) the range of the binding region in the chromosome, (xi) TargetScan prediction outcome, (xii) PITA computed ΔΔE, (xiii) miRanda computed binding energy and (xiv) miRanda computed score.

### Accessibility

The database can be accessed by the following link:

https://data.cyverse.org/dav-anon/iplant/home/corocla/MBS.bed

and can be loaded into UCSC genome browser as custom track. Other genome browsers use similar approaches, but the loading of the database has been tested only in UCSC genome browser. Html external files are accessible through the UCSC detail web pages. The csv file is available at the link:

https://data.cyverse.org/dav-anon/iplant/home/corocla/MBS_ENST-ENSG-NAME_MIRNA.csv.zip

To have a single-click access to an UCSC full browser configuration containing the MBS track for human assembly GRCh38, please just follow this link to a ‘Saved Session’ in UCSC:

https://genome.ucsc.edu/s/corocla/MBS

A user-friendly web app enables easy filtering of both bedDetail and csv file to retrieve BS related to miRNAs or genes of interest. The web app is available at the link:

https://datasharingada.fondazionerimed.com:8080/MBS

## Case Studies

In order to describe some of the analyses that can be performed with the MBS track, we will explore the miRNA binding site regions predicted for the *LMNA* gene. *LMNA* produces many mature transcripts that code for different isoforms with specific physiopathological roles (21, 22). Our database contains information about 38 *LMNA*-related transcripts.

In our database, we collected 5025 highly predicted miRNA interactions in the *LMNA*-related transcripts, resulting in 612 BS in the MBS track. BS and the transcripts can be visualized in the UCSC browser as shown in Figure 1. Specifically, the MBS track (pink) shows the localization of the predicted miRNA binding sites, and the GENCODE track shows the *LMNA* transcripts located in the chosen region.

### Comparison of BS in different transcripts of the same gene

Due to their different composition in terms of exons, different transcripts of the same gene can show different miRNA binding site predictions. Indeed, when a predicted binding site is located within an exon, if that exon is not contained in the mature transcript, the binding site is missed by canonical predictions. As an example, in the region chr1:156138774–156138792, two miRNAs (hsa-miR-10394-5p and hsa-miR-1205) are predicted to bind two transcripts out of the 38 *LMNA* transcripts. The information about each miRNA/transcript interaction is reported in a dedicated html page, an example of which is shown in Figure 2. The missing transcripts do not contain the exon where the BS is located; therefore, the BS cannot be associated to those transcripts. In addition, the different composition in terms of exons affects the binding energies computed by the BS prediction algorithms, especially the ΔΔE energy computed by PITA. In fact, the different exon composition can affect the BS accessibility, resulting in differences in the computed binding scores.

### The case of the aberrant splicing site involved in the Hutchinson-Gilford progeria syndrome

MBS track can be of great aid in studying the effects of mutations and/or alternative splicing on the landscape of miRNA bindings. The case study selected is the Hutchinson-Gilford progeria syndrome (HGPS). HGPS is a human disease of genetic origin (MIM number 177670). HGPS causes a premature aging phenotype with peculiar epigenetic features (23). HGPS is usually caused by specific mutations around a splicing site within the *LMNA* gene that induces an increased rate of aberrant spicing events that in turn lead to the accumulation of a pathogenic protein (namely progerin). Figure 1 shows how MBS track, together with other tracks embedded in UCSC, can help studying the possible effects of mutations that causes HGPS on miRNA binding sites. The vast majority of the causative mutations for HGPS cluster in a specific genomic region, indeed the canonical form of HGPS, are caused by the C1824T mutation (OMIM allelic variant phenotype 150330.0022 as shown in Figure 1a). Those mutations cause the production of a splicing form lacking 150 nucleotides, marked by a red star in Figure 1 (NM_001282626.2). Indeed, MBS track allows to observe, with a user-friendly approach, that hsa-miR-330-5p (the blue arrow in Figure 1) is predicted to recognize specifically the aberrant splicing form involved in HGPS only, while the potential regulation of a set of predicted miRNAs on the same form is abrogated (Figure 1b). The use of MBS track allows to perform analyses that would not be possible (or far less straightforward) with existing tools. Indeed, the information here reported was lost in previous researches that specifically investigated the role of miRNAs in *LMNA* regulation and HGPS (24, 25).

## Discussion

The analysis of miRNAs functions is not an easy task. The lack, with few exceptions (6), of efficient high throughput methods to retrieve experimentally validated miRNA binding sites over the whole transcriptome leads the scientific community to rely on prediction algorithms. Prediction tools are usually used for a specific miRNA and/or a specific gene, and very often, the context in which the binding can occur is lost to the investigator.

MBS database and annotation custom track try to make the access to the information about the miRNA bindings in human easy, allowing the user to retrieving the information needed without losing the information about the context, all in a user-friendly access, browsing and interfacing.

The first step for setting up the MBS track has been to populate the database with high-confident miRNA binding sites. This has been done by leveraging three different algorithms widely used by the scientific community (7, 8, 13–15) and retrieving only the binding sites predicted by all three algorithms with a sufficient score of confidence. The use of multiple algorithms is a strategy previously used by many researchers to reduce the possibility of false positives (7, 8, 25, 26).

With the aim to reduce the biases in the analyses, the predictions have been run not only for the 3ʹUTRs of mRNAs that are considered the hot spots for miRNA binding but also for all the length of transcripts: many pieces of evidence (27–29) suggest that every portion of the mature transcripts can be targeted by miRNA-containing complexes to exert their regulatory functions. This is one of the differences with some other previous approaches (13, 15, 30).

Another feature of MBS is that it takes into account the different transcripts arising from the same gene. Many databases recollect the predicted or validated binding sites on a per gene classification (30, 31). In eukaryotes, and specifically in humans, each gene can produce different mature transcripts, both for alternative transcriptional starting site and/or for alternative splicing events. Considering that alternative splicing is one of the main regulatory mechanisms of the cell and that alternative splicing can produce mature messengers that code for proteins with radical different activities, MBS can be a tool of the greatest utility when analyzing genes prone to alternative splicing. Indeed, in human, a vast majority of genes undergo alternative splicing. Noteworthy, alternative splicing can produce mature mRNAs that have the same coding sequences but possesses different 3ʹUTR that, as stated before, is considered the main hub for miRNA binding and the following regulation. For example, this kind of regulation is central for the activities of *LMNA* gene that produces nuclear lamin A and nuclear lamin C by alternative splicing and whose 3ʹUTR are different; the differential binding of miRNAs to these 3ʹUTRs has been proposed to play a central role in the HGPS phenotype (23). Alternative splicing has also a recognized role in cancer transformation, where splicing events and riboproteic complexes involved in splicing are usually subverted (32–36). One of the greatest advantages of MBS track, when visualized in a genome browser, is the ease with which differential miRNA binding to different transcripts of the same gene can be identified. In the same way, miRNA that requires a proper exon–exon junction to bind can be easily identified as a spliced alignment in the graphical interface of the genome browser. Indeed, many human diseases can be caused by alteration of proper exon–exon junction and exon junction complex functions (37), and MBS provides an easy interface to study this aspect of mRNA maturation in the light of miRNA binding events.

MBS track, when visualized in a genome browser, can be used in concert with other tracks, both embedded in the genome browser itself and customized. Thanks to this, the researcher has the opportunity to integrate information about miRNA binding prediction with other information needed in the study. From this perspective, the utility of MBS matches the level of customization that is indeed quite great in UCSC.

Moreover, for the researcher interested in the details of the prediction, a link is generated in the browser for each binding site to a static external web page where the information of the predictions is stored in great details.

In brief, the user can browse to the gene(s) or region(s) of interest and quickly find the miRNA binding sites for all the transcripts, comprehensive of alternative binding, and the user can integrate this information with all the other information needed. All these features are integrated in a friendly interface extremely easy to use. MBS has been conceived as platform to aid the study of miRNA binding sites in human avoiding the risk of losing information of the genomic context.

## Limitations and Future Development

MBS track does not revolutionize the field. It is just conceived as an extremely useful tool for researchers that study miRNA biology in the contest of the human genome and transcriptome. To our knowledge, a somehow similar UCSC track has been developed in ref (38), but it is available for hg19 only. A planned future development is to integrate more information in the ‘detail pages’ and add a more pleasant interface than basic html. Another important development *in fieri* is to add the possibility to retrieve the information about binding predictions more dynamically, allowing the user to select the thresholds for the three algorithms used. We also plan to develop machine learning–based algorithms to optimize the predictions of miRNA targets, with an approach focused on the features describing each single binding site. At least, we are also planning to extend MBS to other genome assemblies.

## Conclusion

MBS annotation track, when used in a genome browser such as UCSC, will be a useful tool for researcher interested in studying miRNA binding sites in the genomic context without losing the information about how alternative splicing affects the binding itself. Moreover, MBS can be navigated in a friendly interface extremely easy to use. MBS has the potential to be of great support to researchers in the field.

## Data Availability

BedDetail file URL: https://data.cyverse.org/dav-anon/iplant/home/corocla/MBS.bed CSV file URL: https://data.cyverse.org/dav-anon/iplant/home/corocla/MBS_ENST-ENSG-NAME_MIRNA.csv.zip Single-click access to UCSC full browser configuration containing the MBS track for Human assembly GRCh38: https://genome.ucsc.edu/s/corocla/MBS Web app to query the database: https://datasharingada.fondazionerimed.com:8080/MBS
